# Participation of androgen and its receptor in sex determination of an amphibian species

**DOI:** 10.1371/journal.pone.0178067

**Published:** 2017-06-05

**Authors:** Akira Oike, Maho Kodama, Shigeki Yasumasu, Takashi Yamamoto, Yoriko Nakamura, Etsuro Ito, Masahisa Nakamura

**Affiliations:** 1 Department of Biology, Faculty of Education and Integrated Arts and Sciences, Waseda University, Shinjuku-ku, Tokyo, Japan; 2 Department of Materials and Life Sciences, Faculty of Science and Technology, Sophia University, Chiyoda-ku, Tokyo, Japan; 3 Department of Mathematical and Life Sciences, Graduate School of Science, Hiroshima University, Higashi-Hiroshima, Hiroshima, Japan; 4 Department of Science Education, Faculty of Education, Ehime University, Matsuyama, Ehime, Japan; University of Hyderabad, INDIA

## Abstract

**Introduction:**

In the Japanese frog *Rana* (*R*.) *rugosa* the androgen receptor (*AR*) gene on the W chromosome (*W*-*AR*) is barely expressed. Previously we showed that incomplete female-to-male sex-reversal occurred in *Z-AR* transgenic female frogs. To date, however, there is no report showing that AR with androgens can determine genetically programed male sex fate in any vertebrate species. Here, we examined whether AR together with androgens functions as a sex determinant in an amphibian species.

**Methods:**

To examine whether complete female-to-male sex-reversal occurs in *R*. *rugosa* frogs, we produced *AR*-transgenic (Tg) and -knockdown (KD) female *R*. *rugosa* frogs by the *I-SceI* meganuclease-mediated gene trap and CRISPR/Cas9 system, respectively. *AR*-Tg and -KD tadpoles were reared in water containing testosterone (T) at 0 to 7.1 ng/ml. Frozen sections were prepared from the gonads of metamorphosed frogs and immunostained for laminin, Vasa, Pat1a, CYP17 and AR. We also employed PCR analysis to examine *Dmrt1*, *Pat1a* and *CYP17* expression in the gonads of KD and placebo-KD female frogs.

**Results:**

Complete female-to-male sex-reversal occurred in the *AR*-Tg ZW female frogs when a low dosage of T was supplied in the rearing water of tadpoles. However, no sex-reversal was observed in *AR*-KD ZW female frogs when the gonads were treated with dosages of T high enough to induce complete female-to-male sex-reversal even in wild type frogs.

**Discussion:**

These results suggest that AR with its androgen ligand functions as a male sex-determinant in the ZW type *R*. *rugosa* frogs.

## Introduction

In most vertebrate species sex is genetically determined. As in other vertebrate species, heterogametic sex chromosomes in amphibians determine the male (XX/XY) or female (ZZ/ZW) fate [1]. Of great interest in this regard, the Japanese *R*. *rugosa* frog possesses two sex-determining systems (the XX/XY and ZZ/ZW types) within the same species [2]. The frogs of the East, West and Central Japan populations are male heterogametic, whereas those of the North are female heterogametic [3]. In addition, frogs in the North and Central zones have heteromorphic sex chromosomes, whereas those in the East and West carry the homomorphic type [3]. To date, however, no sex-determining gene has been found in this species.

Androgens exert various effects in male reproductive organs, brain skeletal muscle and other target tissues. Androgenic effects are mediated by tissue-specific transcriptional control of target genes via the nuclear androgen receptor (AR) [4]. In *AR*-knockout mice, males have a female-like appearance and body weight, but female-to-male sex-reversal does not occur [5, 6]. Therefore, the *AR* does not appear to participate in sex determination in mice, although the *Sry* on the Y chromosome has been identified as a male sex-determinant in this species [7].

In the *R*. *rugosa* frog the *AR* gene is located on the sex (X, Y, W and Z) chromosomes, reportedly on the inverted region of the Y and W chromosomes [8]. Structural rearrangements such as inversions, translocations and deletions are known to cause degradation of native genes by accumulation of deleterious mutations [9, 10]. Thus, it is likely that the *AR* gene is undergoing a process of evolutional degradation due to the lack of recombination between the inverted and non-inverted regions of the sex chromosomes (X vs. Y, and Z vs. W). It is known that the *AR* gene on the W chromosome (*W*-*AR*) is hardly expressed in *R*. *rugosa* embryos, perhaps due to variations in promoter element and cognate transcription factor interaction between the *W-* and *Z-AR* genes [10]. However, when *W*-*AR* is transgenically expressed in *Xenopus* A6 cells, W-AR proteins can trans-activate androgen-dependent transcription in reporter assays [10], indicating that *W-AR* degradation is incomplete in *R*. *rugosa*. Interestingly, up-regulation of the *AR* expression is observed in the male gonad of *R*. *rugosa* tadpoles prior to sex determination [10]. Moreover, male gonads of *R*. *rugosa* synthesize more androgens than females [11]. In view of these findings, we hypothesized that the *AR* could be involved in male sex determination in this species. To address this issue, we produced *Z*-*AR*-transgenic (Tg) female (ZW) frogs. A subset of these frogs developed a hybrid of testis and ovary, called ovotestis. In other words, incomplete female-to-male sex-reversal took place in Tg ZW frogs. It is known that the AR protein contains an N-terminal trans-activation domain (NTD), a central DNA binding domain (DBD), and a C-terminal ligand binding domain (LBD) that binds androgens such as testosterone (T) and dihydrotestosterone [12]. Thus, we speculated that incomplete sex-reversal would come about from limited levels of androgen in the gonad of ZW frogs. To examine the possibility that AR together with androgens could determine genetically programed male sex in *R*. *rugosa*, we produced *AR*-knockdown (KD) ZW frogs, as well as Tg ZW frogs. By employing the CRISPR/Cas9 system [13], we successfully mutated the *AR* gene lacking the NTD, DBD and LBD by frame-shifting. Here we report for the first time that AR together with androgens can be a male sex-determinant in a vertebrate species.

## Materials and methods

### Ethics statement

All the animal experiments in this study were performed with official approval from the Committee of Animal Experimentation of Waseda University (Permit Number: 2016-A076) as described in detail previously [3].

### Animals and determination of gonad lengths

For all experiments the ZZ/ZW type *R*. *rugosa* was used. Unfertilized eggs were artificially ovulated and inseminated [13]. Fertilized eggs were developed to tadpoles one week after the stage (St.) 25 and transferred into water containing various concentrations of testosterone (T) (ASKA Pharmaceutical Co., Ltd., Tokyo, Japan) to induce female-to-male sex-reversal. Frogs, one week after metamorphosis, were sacrificed to remove gonads surgically for histological and PCR analyses. Embryos and tadpoles were staged according to Shumway [14] and Taylor and Kollros [15]. The genetic sex of each tadpole was determined at the molecular level as previously reported [16]. Photographic images of external appearance of gonads were taken and the length of each gonad was measured.

### Total RNA preparation and cDNA synthesis

Total RNA was prepared from the gonads of frogs and then cDNA was synthesized to use for PCR analysis as previously reported [16].

### Construction of *AR*-expression vector

We constructed the vectors for transgenesis as reported in detail elsewhere [3].

### Production of Tg frogs

We produced Tg ZW frogs, using the *I-SceI* meganuclease-mediated gene trap [3]. Fertilized eggs were injected with *I-Sce*I meganuclease (NEB, Tokyo, Japan) and the *I-Sce*I-cleaved plasmid encoding Z-AR and V5 (GKPIPNPLLGLDST), using a NANOJECT II injection apparatus (Drummond, Broomall, PA, USA). We also produced the placebo-Tg (pTg) ZW frogs as control by injecting *I-Sce*I-cleaved pDPCG vectors and the meganuclease into ZW embryos. Tg and pTg embryos and tadpoles were reared as previously described [3]. To confirm *Z-AR* integration into genomic DNA, we extracted DNA from the tail tip of all Tg tadpoles at St. V, using the AllPrep DNA/RNA Micro Kit (QIAGEN, Tokyo, Japan) [10]. The PCR primers used were: forward, 5’-GCGGTTTTTCCAACTTACCA-3’ and reverse, 5’-CGAGACCGAGGAGAGGGTTA-3’.

### Transgene expression in Tg gonads

We employed PCR analysis to examine *Z-AR/V5* expression in Tg ZW gonads. Total RNA was prepared from the gonads of wild-type (Wt) and Tg frogs just after metamorphosis using ISOGEN (NIPPON GENE, Tokyo, Japan) and cDNAs were synthesized [16]. The PCR reaction consisted of 4 min at 94°C, followed by 35 (*Z-AR*/*V5*, *Dmrt1* and *CYP17*) of 95°C (30 sec), 62°C (30 sec), and 72°C (1 min), ending with 7 min of extension at 72°C. DNA fragments for *Z-AR*/*V5* (260-bp), *CYP17* (330bp, AB284119), and *Dmrt1* (374-bp, AB272609) cDNAs were amplified by PCR using a set of primers for each respective template. Primer sequences are given in Table 1.

**Table 1:** Primer sequences for PCR analysis.

*Dmrt1*	F	5’-CCATTGAACAAGCCTCGCAAA-3’
	R	5’-TCACTGGCACTGGGTAGAGGAATAG-3’
*Pat1a*	F	5’-CACTTTTGGCATGTACAGCACTTG-3’
	R	5’-TGTCCAACAGCACCCCCCTACG-3’
*Z-AR*/*V5*	F	5’-GCGGTTTTTCCAACTTACCA-3’
	R	5’-CGAGACCGAGGAGAGGGTTA-3’
*CYP17*	F	5’-CGCTGTGTATGTTCGGTGAAGG-3’
	R	5’-GGTCTCGAGCTGCCACTGACT-3’
*GA3PDH*	F	5’-GAAGTGAAGGCTGACGGAGGA-3’
	R	5’-CGCCTTGTCATAGCTTTCATGGT-3’

F; Forward. R; Reverse

### *AR* gene mutagenesis

*Cas9* cDNA in the pCS2+ vector was obtained from Thermo Fisher Scientific Inc. (Yokohama, Japan). The DNA construct was linearized with *Not*I and transcribed with the mMessage mMachine SP6 Kit (Thermo Fisher Scientific Inc.) to produce capped *Cas9* mRNA, which was then purified with the RNeasy Mini Kit according to the RNA clean protocol (QIAGEN). To create an engineered single guide RNA (gRNA) expression vector, we placed a T7 promoter followed by two *Bsa*I sites upstream of the recently described gRNA scaffold [13]. The gRNA was designed to target protospacer sequences in the *AR* gene of interest with the form 5’-CC-(N)20-GGG-3’. The GGG was the protospacer-adjacent motif (PAM). The resulting construct was digested with *Dra*I and transcribed using the mMessage mMachine T7 Kit (Thermo Fisher Scientific Inc.). The gRNA was purified using the RNeasy Mini Kit (QIAGEN). We produced KD ZW frogs using the CRISPR/Cas9 system by which we have successfully disrupted the NTD, DBD and LBD of the *AR* gene by frame-shift.

### Analysis of KD gonads

We injected *Cas9* mRNA at a dose of 500 pg per embryo together with gRNA (300 pg/embryo) targeting the *AR* into 2202 one-cell stage *R*. *rugosa* embryos. The MMR (Marc’s Modified Ringer) buffer (4.6 nl, pH 7.8) and gRNA (300 pg/embryo) were also injected into 1071 embryos as control. We designated the placebo *AR*-knockdown embryos as “pKD” embryos. After KD and pKD embryos were subjected to genomic PCR to amplify the target region, PCR products were inserted into a pTAC-2 vector using a TA PCR Cloning Kit (BioDynamics Lab. Inc., Tokyo, Japan) and sequenced.

### Immunohistochemistry

Immunohistochemistry was performed as previously described [17]. The primary antibodies against Vasa, Pat1a, CYP17 and AR were prepared in our laboratory [3, 18] and the anti-laminin antibody was purchased from Sigma-Aldrich (St. Louis, MO, USA). Frozen sections from Wt ZZ testes and Wt ZW ovaries, and Tg, pTg, KD and pKD ZW gonads were stained with the laminin antibody at a dilution of 1:300, Vasa at 1:1,000, Pat1a at 1:1,000, CYP17 at 1:2,000 and AR at 1:2,000. This was followed by the secondary goat anti-rabbit IgG Alexa Fluor 488 antibody (for laminin, Vasa and Pat1a) and goat anti-mouse IgG Alexa Fluor 555 antibody (for CYP17 and AR) (Life Technologies, Tokyo, Japan) or non-immune serum. Positive signals were detected under a fluorescence microscope (Nikon, model ECLIPSE E600, Tokyo, Japan).

### Expression of genes in KD gonads

PCR analysis was performed to examine the expression of genes in the KD and pKD gonads of *R*. *rugosa* frogs after metamorphosis. The reaction mixture contained 4 μl of master mix, 4 μl of a 2.5 μM concentration of each forward and reverse primer (Table 1), 1 μl of each template cDNA and 11 μl of water in a final volume of 20 μl. *CYP17*, *Dmrt1* and *Pat1a* (LC013336) cDNAs were used as template. *GA3PDH* (AB284116) cDNA was used to standardize the mRNA input levels of each gene. PCR products were electophoresed on 4.5% polyacrylamide gels and stained with ethidium bromide [16]. Band intensity was determined by a software program (Image J ver. 1.49q made by Rasband W, NIH, USA (http://imagej.nih.gov/ij/download.html). Relative expression levels of each gene was calculated by the following formula: relative expression level = gene X (T-treated) x GA3PDH (T-untreated) / gene X (T-untreated) x GA3PDH (T-treated) [16].

### Statistics

Data are represented as the mean±SE. Differences (P<0.05) determined using one-way ANOVA, were considered statistically significant.

### Image acquisition and analysis

Images were scanned and adjusted for brightness and contrast by Adobe Photoshop CS2.

## Results

### Analysis of Tg gonads

#### Histology

**Fig 1:**
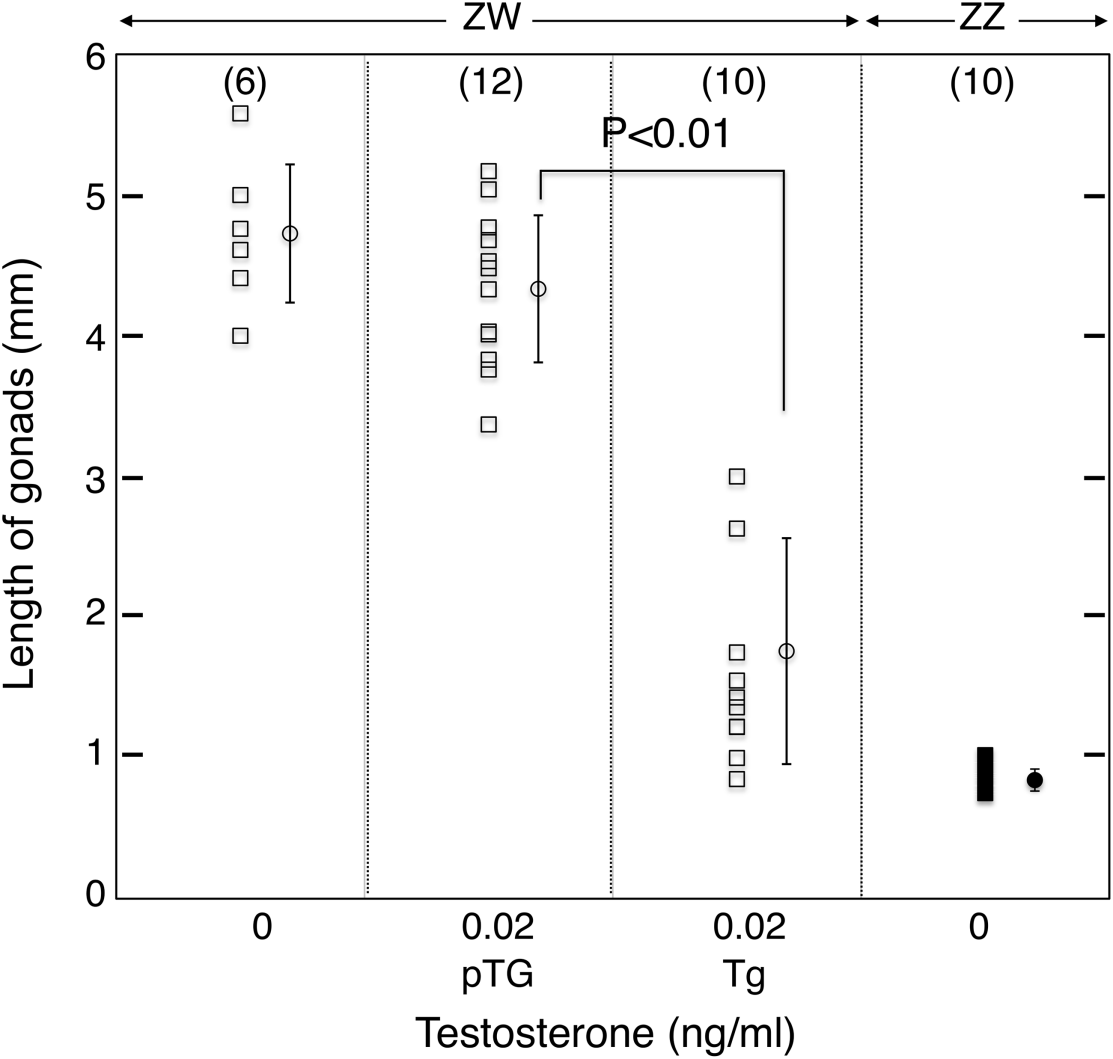
Change in Tg gonad lengths. The number of gonads from different frogs is shown in parentheses at the top of the panel. Each bar represents the mean±SE for gonadal lengths in each group of frogs. ZW, female and ZZ, male.

The wild-type (Wt) ZW ovaries were much bigger than the Wt ZZ testes (4.7±0.5 vs 0.8±0.1 mm in length, respectively; Fig 1). When the ovaries of transgenic pTg ZW frogs were treated with T at 0.02 ng/ml in the rearing water during the tadpole stage, they did not decrease significantly in size when compared to the Wt ZW ovaries (4.3±0.5 vs 4.7±0.5 mm, Fig 1). Previously it has been shown that female-to-male sex-reversal is not induced when an ovary is treated with T at 0.02 ng/ml in rearing water of *R*. *rugosa* tadpoles [16]. However, in the present study, the average sizes of the Tg ZW gonads at 0.02 ng/ml of T became significantly smaller than those of the pTg ZW gonads (Fig 1, 1.6±0.9 vs 4.3±0.5 mm, P<0.01).

#### Expression of the *Z-AR/V5* transgene

**Fig 2:**
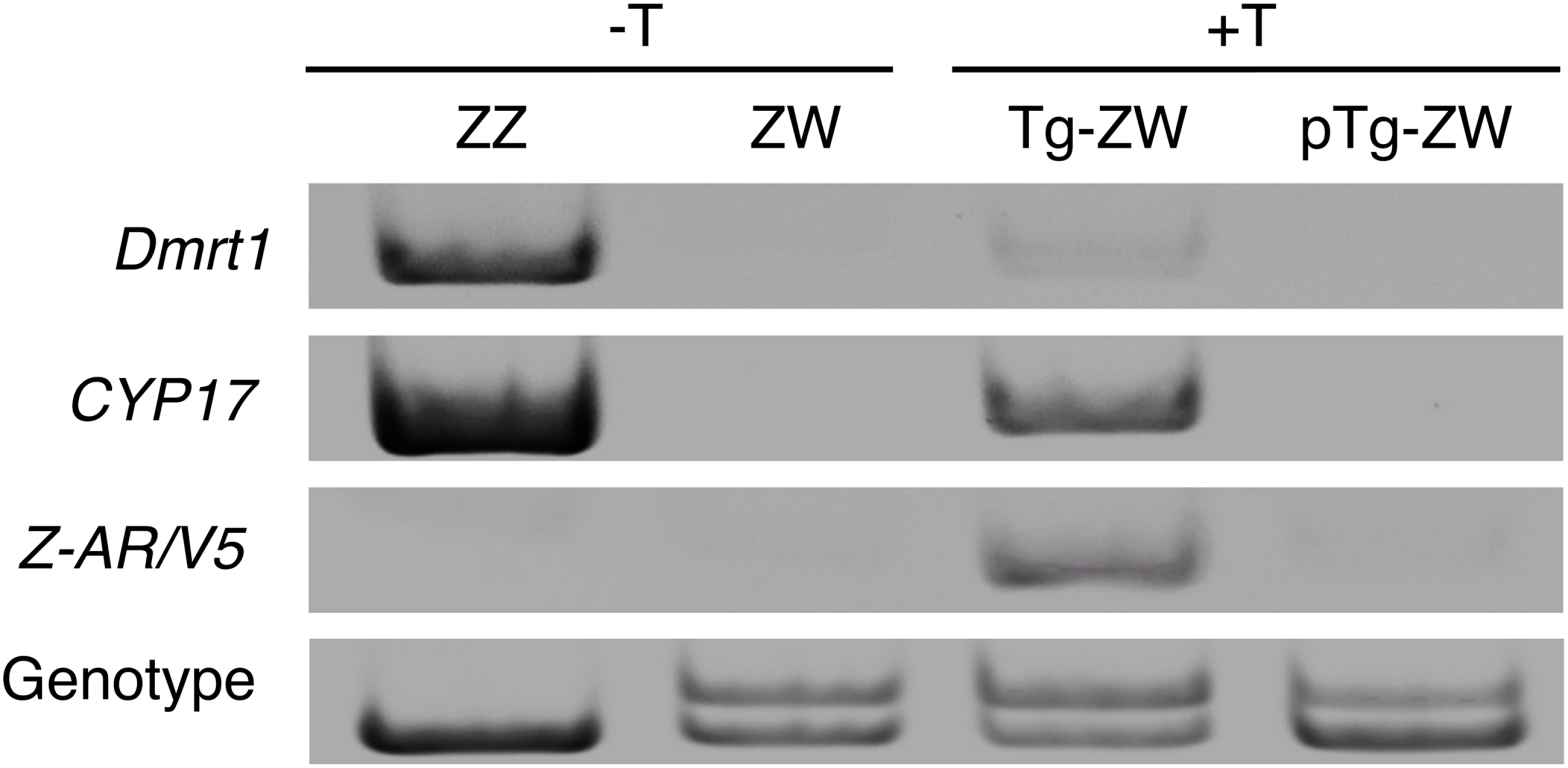
*Z-AR/V5*, *CYP17* and *Dmrt1* mRNA expression. RT-PCR analysis was employed to detect *Z-AR/V5*, *CYP17* and *Dmrt1* mRNA in Wt ZZ and Wt ZW, and Tg ZW and pTg ZW gonads treated with T at 0.02 ng/ml in the rearing water. Top panel, *Dmrt1* expression; 2nd panel, *CYP17* expression; 3rd panel, *Z-AR*/*V5* expression; bottom panel, genetic sex of each frog. ZZ, male and ZW, female.

We examined the expression of the *Z*-*AR/V5* transgene by PCR analysis in 10 Tg ZW frogs. The *Z-AR*/*V5* mRNA was exclusively transcribed in all the Tg ZW gonads, but the result from a representative sample was shown on the third panel of Fig 2.

Expression of the *Dmrt1* and *CYP17* genes required for masculinization of the gonad in *R*. *rugosa* [16] was also examined. *Dmrt1* and *CYP17* mRNAs were expressed at high levels in the Wt ZZ gonad (testis), but not in the Wt ZW gonad (ovary) (Fig 2, first and second panels, respectively). At 0.02 ng/ml of T (+T), these two genes were transcribed in the Tg ZW gonads at significantly higher levels than in the pTg ZW gonads (Fig 2).

#### Immunohistology

**Fig 3:**
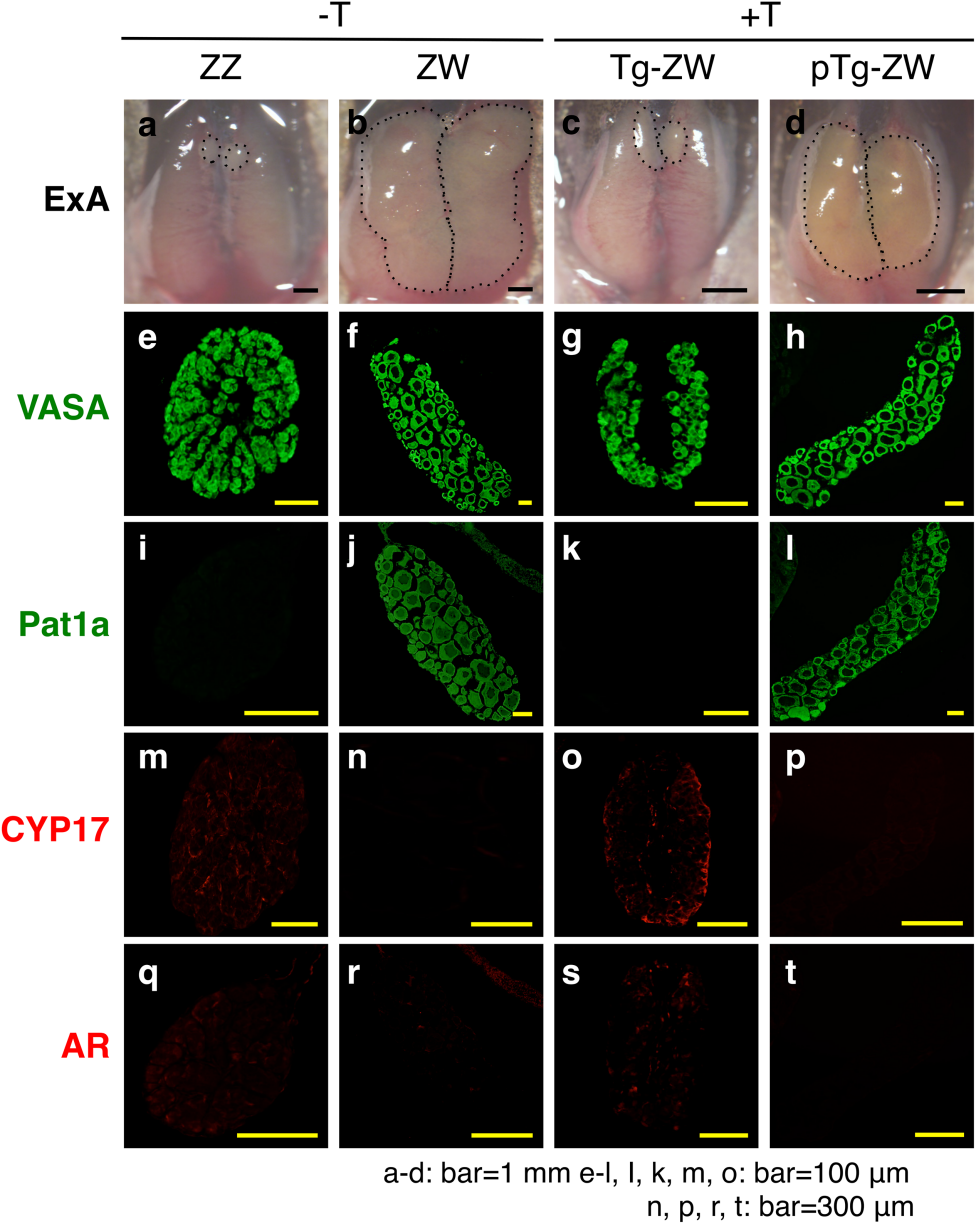
Immunohistology of Tg gonads. Frozen sections (7 μm) from the Tg and pTg ZW gonads treated with (+) and without (-) T at 0.02 ng/ml in the rearing water, and Wt ZZ, Wt ZW, Tg ZW and pTg ZW gonads were prepared and stained for Vasa, Pat1a, CYP17 and AR. ExA, external appearance. ZZ, male and ZW, female.

We prepared frozen sections from the Wt and Tg gonads of frogs and stained for Vasa, Pat1a, CYP17 and AR. Vasa is a protein specific to germ cells in both the male and female gonads, but Pat1a is specific to only the immature oocytes in the ovary [18]. CYP17 is a steroidogenic enzyme responsible for the conversion of progesterone into androstenedione [19]. The size of the ZW ovary appeared much bigger than that of the testis (Fig 3a and 3b). The Tg ZW gonad became a testis when treated with 0.02 ng/ml T (+T) (Fig 3c). However, the pTg ZW gonad developed into an ovary when it was treated with 0.02 ng/ml T (+T) (Fig 3d).

Strong immuno-positive signals for Vasa were observed in all the gonads examined: Wt testis (Fig 3e), Wt ovary (Fig 3f) as well as Tg (Fig 3g) and pTg (Fig 3h) ZW gonads treated with 0.02 ng/ml T (+T). Positive signals for Pat1a were produced in only the Wt ZW (Fig 3j) and pTg ZW ovaries (Fig 3l), and not in either Wt ZZ testis (Fig 3i) or Tg ZW gonads treated with T (testis) (Fig 3k). On the other hand, CYP17 and AR positive signals were produced in the Wt testis (Fig 3m and 3q) and Tg ZW gonads (+T) (testis) (Fig 3o and 3s), but were undetectable in Wt ovary (Fig 3n and 3r) and pTg ZW gonads (+T) (ovary) (Fig 3p and 3t). These results clearly indicate that Tg ZW gonads are transformed into testes when T is supplied to the rearing water of tadpoles, but T could not masculinize an ovary into a testis without integration and expression of an exogenous *Z-AR* in the genomic DNA of *R*. *rugosa* females.

### Analysis of KD gonads

#### Gene mutagenesis

**Fig 4:**
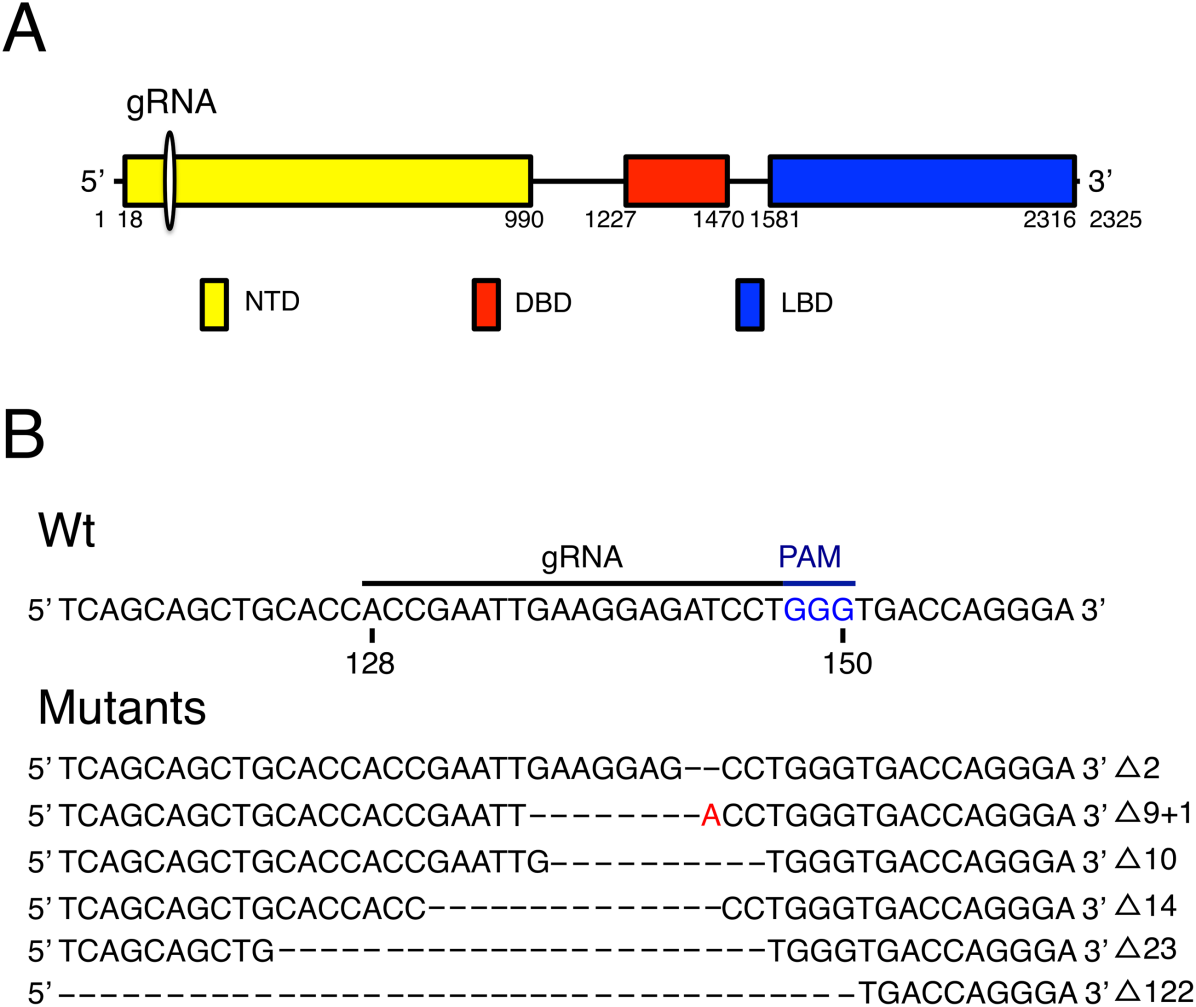
*AR* gene mutagenesis. (A) Three domains of the *AR* gene. The DNA sequencing encoding the 3 functional domains of the androgen protein (NTD, DBD and LBD) are shown by closed boxes. The numbers indicate the nucleotide positions of the domains in the *R*. *rugosa AR* gene (AB372103, https://www.ncbi.nlm.nih.gov/protein/315013360). gRNA indicates the site for mutagenesis at nucleotide positions 114 to 160 of the *Z-AR* cDNA. (B) Mutation profile from one embryo sequenced after cloning. Wt: The nucleotide sequence from positions 114 to 160 of the *Z*-*AR* cDNA. The PAM sequence (blue) is shown together with the target sequence. Mutations: Mutated sequences of targeting *AR* coding region. The letter A in red indicates the mismatch insertion of A in exchange for T.

We produced KD ZW frogs using the CRISPR/Cas9 system by which we have successfully disrupted the NTD, DBD and LBD of the *AR* gene by frame-shift. When we injected *Cas9* mRNA together with gRNA targeting the *AR* into 2202 embryos, and the MMR (Marc’s Modified Ringer) buffer or gRNA alone into 1071 embryos as control, 511 and 419 embryos developed into tadpoles at St. 25-1W, respectively. Then we examined mutagenesis in 44 KD and 38 pKD frogs with normal outward appearance 1 week after metamorphosis. The mutagenesis rate of KD (ZW) frogs was 50 to 100%, whereas pKD (ZW) frogs showed no genomic mutation. Mutated sequences from one representative KD (ZW) sample injected with *Cas9* mRNA and gRNA are shown in Fig 4B. The mutagenesis rate was 75% (6 out of 8 clones sequenced).

#### Histology

**Fig 5:**
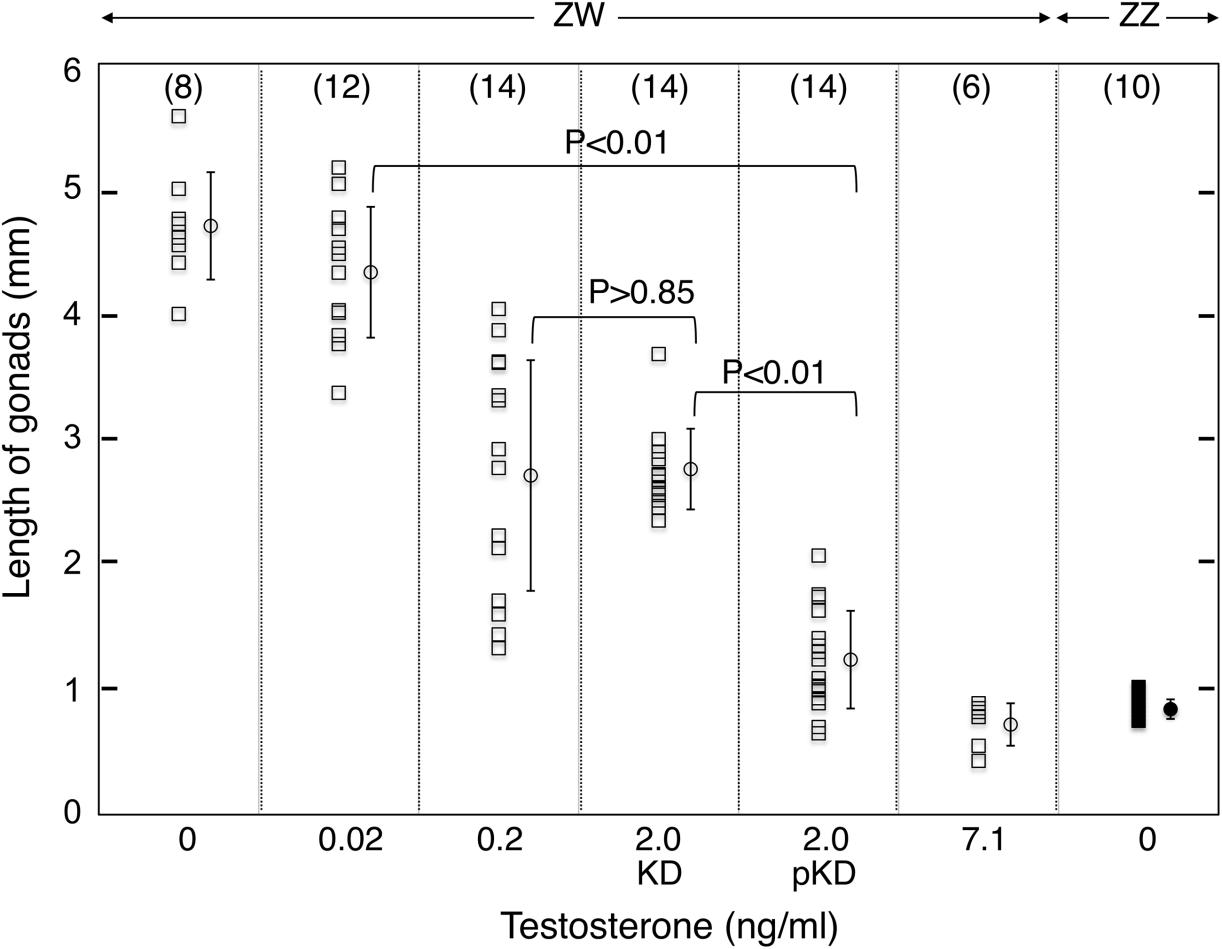
Change in KD gonad lengths. The number of gonads from different frogs is shown in parentheses at the top of each panel. Each bar represents the mean±SE for gonadal lengths in each group of frogs. The mean (±SE) differed from respective groups (P< 0.01), but not significantly from the other group (P>0.85). ZZ, male and ZW, female.

The Wt ZW ovaries were much bigger than the Wt ZZ testes (4.7±0.4 vs 0.8±0.1 mm; Fig 5). When Wt ZW ovaries were treated with T in water (0 to 7.1 ng/ml), ovary size decreased gradually in a dose-dependent manner. The average size of the Wt ZW gonads treated with T at 0.02 ng/ml was similar to that of the Wt ovaries (4.7±0.5 vs 4.3±0.5 mm). The KD ZW gonads treated with 2.0 ng/ml T were similar to those of Wt ZW ovaries treated with T at 0.2 ng/ml (2.8±0.3 vs 2.7±0.9 mm; P>0.85). At 2.0 ng/ml T, all pKD ZW gonads were significantly smaller than the KD ZW gonads (1.2±0.4 vs 2.8±0.3 mm; P<0.01). At 7.1 ng/ml, all Wt ZW gonads were similar in size to the ZZ testes (Fig 5).

#### Immunohistology

**Fig 6:**
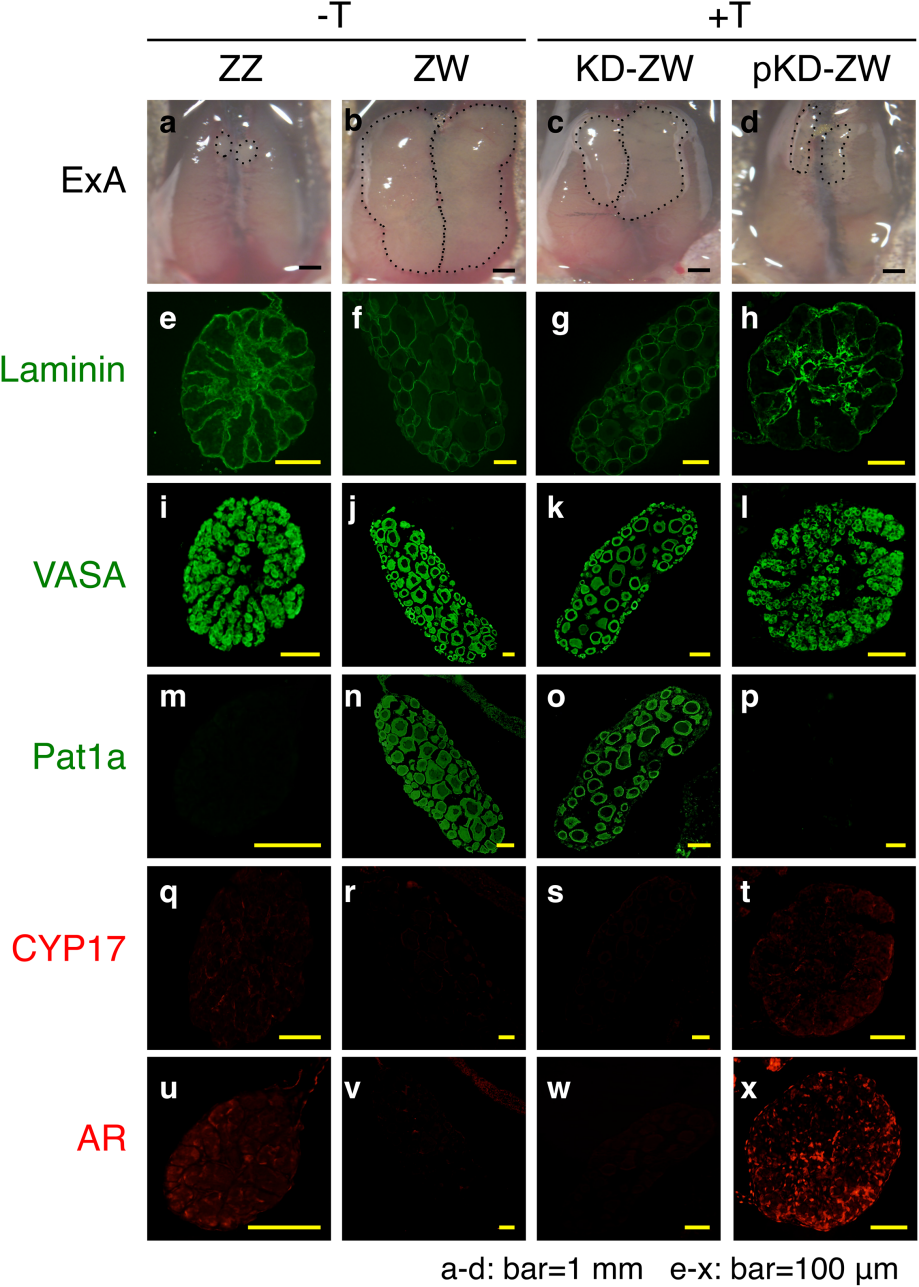
Immunohistology of *AR*-KD gonads. We prepared frozen sections (7 μm) from the Wt ZZ and ZW gonads without T treatment, and KD and pKD ZW gonads treated with (+) and without (-) T at 2.0 ng/ml in the rearing water and stained them for laminin, Vasa, Pat1a, CYP17 and AR. ExA, external appearance. ZZ, male and ZW, female.

We prepared frozen sections from the Wt, Tg, pTg, KD and pKD gonads and immunostained for laminin, Vasa, Pat1a, CYP17 and AR. Laminin is a component of the basement membrane in the male and female gonads [18]. The Wt ZW ovary was much bigger than the Wt ZZ testis (Fig 6a and 6b). The KD ZW gonad was also bigger than the pKD ZW gonad (Fig 6c and 6d). Positive signals for laminin were produced in the basement membrane surrounding the seminiferous tubules in the testis (Fig 6e) and the oocytes in the Wt and KD ZW ovaries (Fig 6f and 6g). This laminin staining delineated the seminiferous tubule structure of the pKD ZW gonads treated with 2.0 ng/ml of T (+T) (Fig 6h). We also observed strong immuno-positive signals for Vasa in the germ cells of both male and female gonads (Fig 6i and 6j) as well as in the KD ZW and pKD ZW gonads (Fig 6k and 6l). Pat1a positive-signals were detected in the oocytes of Wt ZW ovaries (Fig 6n) and KD ZW gonads treated with 2.0 ng/ml of T (+T) (Fig 6o). However, Pat1a signals were not produced by the Wt testis (Fig 6m) or the pKD ZW gonads treated with 2.0 ng/ml of T (+T) (Fig 6p).

CYP17 and AR positive signals were detected in the ZZ testis (Fig 6q and 6u, respectively) and in the pKD ZW gonad treated with 2.0 ng/ml of T (+T) (Fig 6t and 6x). By contrast, positive signals for CYP17 and AR were not produced in the KD ZW gonad treated with T (Fig 6s and 6w) and in the Wt ovary (Fig 6r and 6v). These results indicate that T can transform a Wt ovary into a testis, but this transformation is blocked if the *AR* is knocked down in female *R*. *rugosa* frogs.

#### Gene expression in KD female gonads

**Fig 7:**
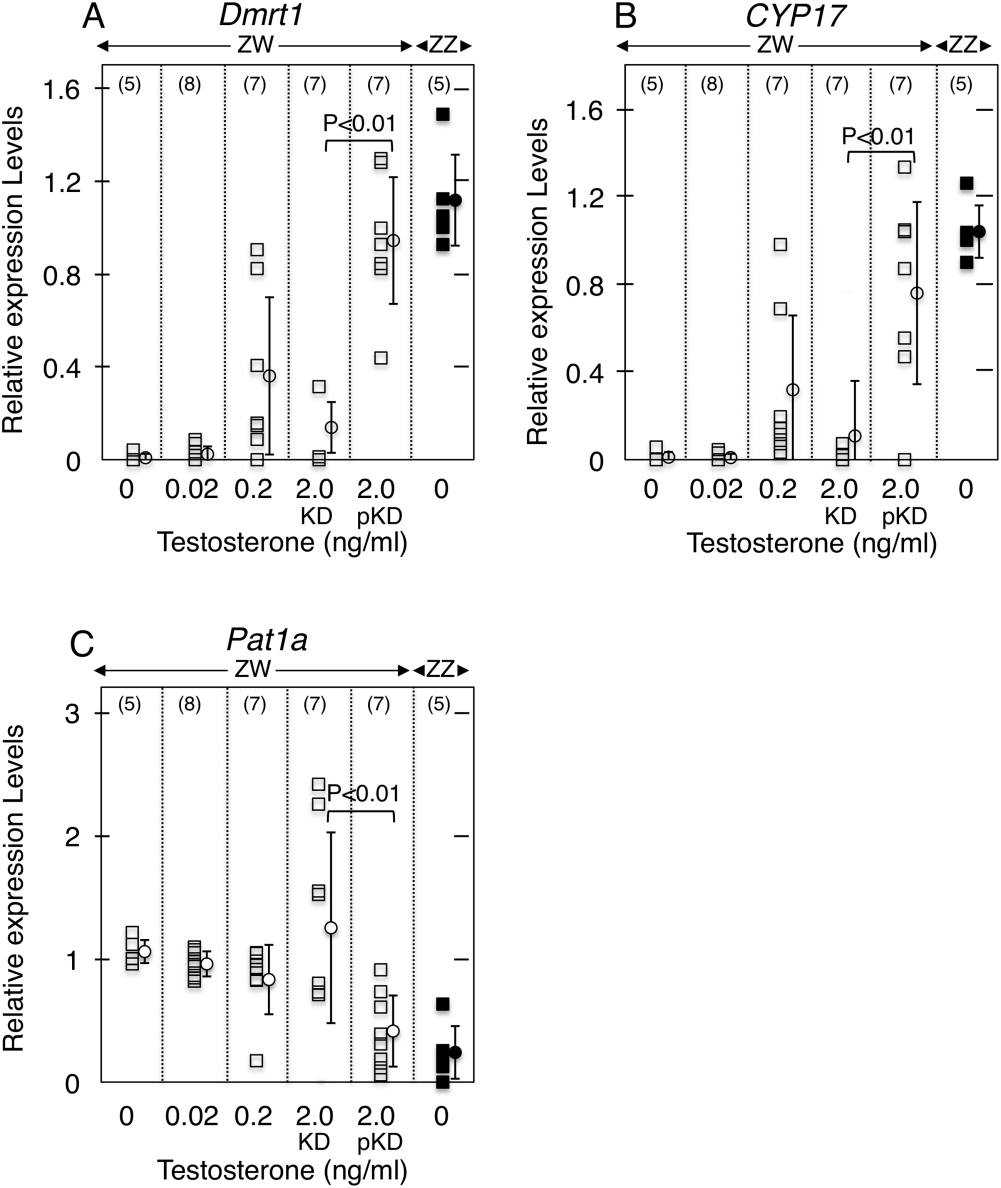
Gene expression in KD gonads. Relative expression levels of *Dmrt1* (A), *CYP17* (B) and *Pat1a* (C) were calculated by the formula given in Materials and Methods. Numbers of the gonads examined are shown in parentheses at the top of each panel. Each bar represents the mean normalized expression (±SE) from the gonads of each group. The mean (±SE) differs from respective groups (P<0.01). ZW, female and ZZ, male.

We reared female tadpoles in water containing T at 0 to 2.0 ng/ml and examined *Dmrt1*, *CYP17* and *Pat1a* expression in the Wt and KD gonads. *Dmrt1* (doublesex and mab-3 related transcription factor 1) is involved in testis formation in *R*. *rugosa* [20]. *Dmrt1* expression was at very low levels in the Wt ZW ovary and the ZW gonad treated with 0.02 ng/ml of T (ovary), but increased in the pKD ZW gonad treated with T at 2.0 ng/ml (testis) (P<0.01, Fig 7A). It was also at low levels in the KD ZW gonad treated with T at 2.0 ng/ml (ovary) (Fig 7A). This was also the case for *CYP17* expression. *CYP17* mRNA expression was high in the Wt ZZ testis (Fig 7B). Its expression increased significantly in the pKD ZW gonad (testis) compared to the KD ZW gonad (ovary) when both were treated with T at 2.0 ng/ml (P<0.01, Fig 7B). At 0 and 0.02 ng/ml of T, levels of *CYP17* expression were low in the ZW gonads (Fig 7B).

Finally, *Pat1a* expression was examined in the Wt ZW and KD ZW gonads. Expression was low in the Wt ZW gonads (Fig 7C). *Pat1a* expression increased significantly in KD gonads treated with T at 2.0 ng/ml (ovary) compared with that in the pKD treated with T at 2.0 ng/ml (testis) (P<0.01, Fig 7C). Previously it has been shown that in *R*. *rugosa* frogs *Pat1a* is specifically expressed in the immature oocytes of the ovary [18], and that complete female-to-male sex-reversal occurs when a Wt ZW ovary is treated with T at 2.0 ng/ml at the tadpole stage [16]. Thus, we can conclude that when the *AR* is knocked-downed in ZW frogs, an ovary will be resistant to transformation into a testis even at T dosages high enough to induce complete female-to-male sex-reversal in Wt *R*. *rugosa* females.

## Discussion

Sex-determining genes have been identified in several vertebrate species: *SRY* in human [21], and *Dmy* [22] and *Sox3* [23] in medaka fish. Each of these genes is located on the Y chromosome and encodes a transcription factor that regulates the expression of a target gene to direct an indifferent gonad toward a testis fate. In addition, *Dmrt1* in chicken on the Z [24] and *DM-W* in *Xenopus* on the W chromosome [25] have been found. Previously we have shown that the *W*-*AR* is hardly expressed in *R*. *rugosa* embryos compared with *Z-AR* [10]. We then hypothesized that *Z*-*AR* came to play a role, perhaps a critical one given its sex hormone-signaling function, in male sex determination in ZZ/ZW *R*. *rugosa*. To clarify this further, we examined whether the *Z-AR* was a critical gene for male sex determination in ZZ/ZW *R*. *rugosa* by analyzing the occurance of sex-reversal in Tg ZW embryos. We observed that a number of the Tg ZW frogs developed varying degrees of masculinized gonad, called ovotestis, indicating that the phenotypic sex of *R*. *rugosa* ZW frogs is partially reversed from female to male if an exogenous *Z-AR* transgene is expressed in the frogs. These findings led us to conclude that the *Z*-*AR* participates in male sex determination in ZZ/ZW *R*. *rugosa*, but is not an exclusive sex determinant in this frog since it encodes a functioning hormone receptor that binds androgens such as T and dihydrotestosterone.

In this study we have successfully produced KD ZW *R*. *rugosa* frogs. This is the first report showing a wild-type frog in which a target gene has been functionally silenced. Previously we have shown that ovaries of *R*. *rugosa* ZW frogs are transformed into testes when treated with T during the tadpole stage [19] and that partial female-to-male sex-reversal occurs in Wt ZW frogs if a *Z-AR* transgene is constitutively expressed [3]. Why doesn’t complete sex-reversal occur in Tg ZW frogs? It may be that insufficient amounts of endogenous T are available to trans-activate the relevant cognate genes, or to initiate the gene cascade necessary for specification of the male fate. Recently, we determined that 0.2 ng/ml in the rearing water is the threshold dosage of T for sex-reversal in *R*. *rugosa* [16]. When Wt ZW tadpoles were allowed to develop into frogs in water containing T at 0.02 ng/ml (one tenth of the dosage), Tg ZW frogs formed testes. This indicates that Tg ZW frogs develops testes when T is supplied in the rearing water during the tadpole stage, but cannot do so if T is not supplied. Sex-reversed testis in the genetically female frogs produce Vasa-positive, but Pat1a-negative signals in germ cells, as in Wt ZZ testis, indicating that the germ cells in the Tg ZW gonads treated with T have been completely masculinized.

Next, pKD ZW frogs developed testes when treated with high dosages of T during the tadpole stage, but KD ZW frogs did not. The latter developed ovaries when treated with high dosages of T, but transformed into testes in the Wt ZW frogs. It should also be mentioned that KD ZZ *R*. *rugosa* frogs examined developed the testes when untreated with high dosages of T during the tadpole stage, which may be due to a low mutagenesis rate. This is supported by the fact that all WW *R*. *rugosa* embryos where the *W-AR* gene is barely expressed become lethal during the tadpole stage [10]. It would be possible that male-to-female sex-reversal occurs in the KD ZZ *R*. *rugosa* frogs if androgen levels in the male gonads became extremely low. Thus, it is evident that AR together with androgens can direct indifferent gonads into testes fate in the ZZ/ZW type *R*. *rugosa*. To the best of our knowledge, this is the first report showing a steroid hormone receptor can function as a male sex determinant in a species of vertebrate.

Previously, we have shown that steroidogenic genes are expressed in the indifferent gonads of both male and female *R*. *rugosa* tadpoles [26]. The *AR* gene is up-regulated in the male gonads prior to sex determination in genetically male *R*. *rugosa* [10] and more androgens are synthesized in *R*. *rugosa* testes during gonadal differentiation than in ovaries [11]. Thus, androgens sufficient for normal male sex determination may be synthesized in the indifferent male gonads of *R*. *rugosa*. Further study is needed to determine the precise levels of androgens in the Wt male and female gonads, quite a challenge due to the small size of tadpole gonads prior to sex determination. Nevertheless, it is clear from the current study that androgen and its receptor play a pivotal role in genotypically programmed male sex determination in this species.

Finally, the *W*-*AR* gene of *R*. *rugosa* seems to have evolved from the proto-type *X*-*AR* of the Korean *R*. *rugosa* [27]. The *W*-*AR* locus is located on the inverted region of the W chromosome of the Japanese *R*. *rugosa* [3, 8] and expressed at extremely low levels in *R*. *rugosa* embryos compared with *Z-AR* [10]. Thus, *Z*-*AR* came to play a role, perhaps a critical one given its sex hormone-signaling function, in male sex determination in ZZ/ZW type *R*. *rugosa*. This report provides evidence for the first time that evolutionary degradation of the *W-AR* gene leads to an ancillary role for *Z*-*AR* together with androgens in male sex determination in an amphibian species.

## Conclusions

Previously we showed that incomplete female-to-male sex-reversal occurred in *Z-AR* transgenic (Tg) ZW females of the *R*. *rugosa* frog. The *AR*-Tg ZW females developed a hybrid of testis and ovary, called “ovotestis.” However, the *AR*-Tg female frogs formed the testes when a low dosage of T was supplied in the rearing water of tadpoles. In the sex-reversed testes the expression of *Dmrt1*, *AR* and *CYP17* genes required for masculinization were significantly up-regulated. Next, we produced *AR*-knockdown (KD) ZW female frogs by the CRISPR/Cas9 system. Interestingly, no sex-reversal was observed in *AR*-KD ZW female frogs when the gonads were treated with dosages of T high enough to induce complete female-to-male sex-reversal, even in wild type frogs. In the *AR*-KD ZW female gonads the expression of genes required for masculinization was not up-regulated. These results indicate that AR together with androgens can be a male sex-determinant in an amphibian species.

## References

[1] Wallace H, Badawy GMI, Wallace BMN. Amphibian sex determination and sex reversal. Cell Mol Life Sci. 1999; 55: 901–909. 10412371 10.1007/s000180050343 PMC11147005

[2] Nishioka M, Hanada H, Miura I, Ruyzaki M. Four kinds of sex chromosomes in *Rana rugosa*. Sci Rep Lab Amphib Biol Hiroshima Univ. 1994; 13: 1–34.

[3] Fujii J, Kodama M, Oike A, Matsuo Y, Min M-S, Hasebe T, et al Involvement of androgen receptor in sex determination in an amphibian species. PLoS ONE. 2014; 9: e93655 10.1371/journal.pone.0093655 24826887 PMC4020753

[4] Sato T, Matsumoto T, Yamada T, Watanabe T, Kawano H, Kato S. Late onset of obesity in male androgen receptor-deficient (ARKO) mice. Biochem Biophys Res Commun. 2003; 300: 167–171. 12480537 10.1016/s0006-291x(02)02774-2

[5] Yeh S, Tsai MY, Xu Q, Mu XM, Lardy H, Huang KE, et al Generation and characterization of androgen receptor knockout (ARKO) mice: An *in vivo* model for the study of androgen functions in selective tissues. Proc Natl Acad Sci USA. 2002; 99: 13498–13503. 10.1073/pnas.212474399 12370412 PMC129702

[6] Kerkhofs S, Denayer S, Haelens A, Claessens F. Androgen receptor knockout and knock-in mouse models. J Mol Endocrinol. 2009; 42: 11–17. 10.1677/JME-08-0122 18923000

[7] Koopman P, Gubbay J, Vivian N, Goodfellow P, Lövell-Badge R. Male development of chromosomally female mice transgenic for *Sry*. Nature. 1991; 351: 117–121. 10.1038/351117a0 2030730

[8] Uno Y, Nishida G, Oshima Y, Yokoyama S, Miura I, Matsuda Y, et al Comparative chromosome mapping of sex-linked genes and identification of sex chromosomal rearrangements in the Japanese wrinkled frog (*Rana rugosa*, Ranidae) with ZW and XY sex chromosome systems. Chromosome Res. 2008; 16: 637–647. 10.1007/s10577-008-1217-7 18484182

[9] Steinemann S, Steinemann M. Y chromosome: borne to be destroyed. BioEssays. 2005; 27: 1076–1083. 10.1002/bies.20288 16163733

[10] Yokoyama S, Oshima Y, Tokita J, Suda M, Shinozuka T, Nakamura M. Androgen Receptor of the Frog *Rana rugosa*: Molecular Cloning and Its Characterization. J Exp Zool. 2009; 311A: 1–17. 10.1002/jez.568 19722274

[11] Sakurai N, Maruo K, Haraguchi S, Uno Y, Oshima Y, Tsutsui K, et al Immunohistochemical detection and biological activities of CYP17 (P450c17) in the indifferent gonad of the frog *Rana rugosa*. J Steroid Biochem Mol Biol. 2008; 112: 5–12. 10.1016/j.jsbmb.2008.07.002 18675354

[12] Shen HC, Shanmugasundaram K, Simon NI, Cai C, Wang H, Chen S, et al In Silico Discovery of Androgen Receptor Antagonists with Activity in Castration Resistant Prostate Cancer. Mol Endocrinol. 2012; 26: 1836–1846. 10.1210/me.2012-1222 23023563 PMC3487628

[13] Guo X, Zhang T, Hu Z, Zhang Y, Shi Z, Wang Q, et al Efficient RNA/Cas9-mediated genome editing in *Xenopus tropicalis*. Development. 2014; 141: 707–714. 10.1242/dev.099853 24401372

[14] Shumway W. Stages in the normal development of *Rana pipiens*. I. External form. Anat Rec. 1940; 78: 139–147.

[15] Taylor AC, Kollros JJ. Stages in the normal development of *Rana pipiens* larvae. Anat Rec. 1946; 94: 7–23. 21013391 10.1002/ar.1090940103

[16] Oike A, Kodama M, Nakamura Y, Nakamura M. A threshold dosage of testosterone for female-to-male sex-reversal in *Rana rugosa* frogs. J Exp Zool. 2016; 325A: 532–538. 10.1002/jez.2037 27677985

[17] Saotome K, Hayashi K, Adachi N, Nakamura Y, Nakamura M. Structural changes in gonadal basement membranes during sex differentiation in the frog *Rana rugosa*. J Exp Zool. 2010; 313A: 369–380. 10.1002/jez.607 20535767

[18] Nakamura Y, Iwasaki T, Umei Y, Saotome K, Nakajima Y, Kitahara K, et al Molecular cloning and characterization of oocyte-specific Pat1a in *Rana rugosa* frogs. J Exp Zool. 2015; 323A: 516–526. 10.1002/jez.1938 26136381

[19] Iwade R, Maruo K, Okada G, Nakamura M. Elevated expression of *P450c17* (*CYP17*) during testicular formation in the frog. Gen Comp Endocrinol. 2008; 155: 79–87. 10.1016/j.ygcen.2007.02.032 17434514

[20] Shibata K, Takase M, Nakamura M. The *Dmrt1* expression in sex-reversed gonads of amphibians. Gen Comp Endocrinol. 2002; 127: 232–241. 12225764 10.1016/s0016-6480(02)00039-4

[21] Sinclair AH, Berta P, Palmer MS, Hawkins JR, Griffiths BL, Smith MJ, et al A gene from the human sex-determining region encodes a protein with homology to a conserved DNA-binding motif. Nature. 1990; 346: 240–244. 10.1038/346240a0 1695712

[22] Matsuda M, Nagahama Y, Shinomiya A, Sato T, Matsuda C, Kobayashi T, et al *DMY* is a Y-specific DM-domain gene required for male development in the medaka fish. Nature. 2002; 417: 559–563. 10.1038/nature751 12037570

[23] Takehana Y, Matsuda M, Myosho T, Suster ML, Kawakami K, Shin-I T, et al Co-option of Sox3 as the male-determining factor on the Y chromosome in the fish *Oryzias dancena*. Nature Commun. 2014; 5: 4157 10.1038/ncomms5157 24948391

[24] Smith CA, Roeszler KN, Ohnesorg T, Cummins DM, Farlie PG, Doran TJ, et al The avian Z-linked gene DMRT1 is required for male sex determination in the chicken. Nature. 2009; 461: 267–271. 10.1038/nature08298 19710650

[25] Yoshimoto S, Okada E, Umemoto H, Tamura K, Uno Y, Nishida-Umehara C, et al A W-linked DM-domain gene, *DM-W*, participates in primary ovary development in *Xenopus laevis*. Proc Natl Acad Sci USA. 2008; 105: 2469–2474. 10.1073/pnas.0712244105 18268317 PMC2268160

[26] Maruo K, Suda M, Yokoyama S, Oshima Y, Nakamura M. Steroidogenic gene expression during sex determination in the frog, *Rana rugosa*. Gen Comp Endocrinol. 2008; 158: 87–94. 10.1016/j.ygcen.2008.04.019 18550057

[27] Ogata M, Lee JY, Kim S, Ohtani H, Sekiya K, Igarashi T, et al The prototype of sex chromosomes found in Korean populations of *Rana rugosa*. Cytogenet Genome Res. 2002; 99: 185–193. 12900563 10.1159/000071592

